# Zidovudine in synergistic combination with nitrofurantoin or omadacycline: *in vitro* and in murine urinary tract or lung infection evaluation against multidrug-resistant *Klebsiella pneumoniae*

**DOI:** 10.1128/aac.00344-24

**Published:** 2024-08-28

**Authors:** Ping Tian, Qing-Qing Li, Ming-Juan Guo, Yun-Zhu Zhu, Rong-Qing Zhu, Ya-Qin Guo, Yi Yang, Yan-Yan Liu, Liang Yu, Ya-Sheng Li, Jia-Bin Li

**Affiliations:** 1 Department of Infectious Diseases & Anhui Center for Surveillance of Bacterial Resistance, The First Affiliated Hospital of Anhui Medical University, Hefei, China; 2 Anhui Province Key Laboratory of Infectious Diseases and Institute of Bacterial Resistance, Anhui Medical University, Hefei, China; 3 Department of Hepatology, The First Affiliated Hospital of Jilin University, Changchun, China; University of Pittsburgh, Pittsburgh, Pennsylvania, USA

**Keywords:** drug repurposing, *Klebsiella pneumoniae*, zidovudine, nitrofurantoin, omadacycline

## Abstract

Limited treatment options and multidrug-resistant (MDR) *Klebsiella pneumoniae* present a significant therapeutic challenge, underscoring the need for novel approaches. Drug repurposing is a promising tool for augmenting the activity of many antibiotics. This study aimed to identify novel synergistic drug combinations against *K. pneumoniae* based on drug repurposing. We used the clinically isolated GN 172867 MDR strain of *K. pneumoniae* to determine the reversal resistance activity of zidovudine (AZT). The combined effects of AZT and various antibiotics, including nitrofurantoin (NIT) and omadacycline (OMC), were examined using the checkerboard method, growth curves, and crystal violet assays to assess biofilms. An *in vitro* combination activity testing was carried out in 12 isolates of *K. pneumoniae. In vivo* murine urinary tract and lung infection models were used to evaluate the therapeutic effects of AZT + NIT and AZT + OMC, respectively. The fractional inhibitory concentration index and growth curve demonstrated that AZT synergized with NIT or OMC against *K. pneumoniae* strains. In addition, AZT + NIT inhibited biofilm formation and cleared mature biofilms. *In vivo*, compared with untreated GN 172867-infected mice, AZT + NIT and AZT + OMC treatment decreased colony counts in multiple tissues (*P* < 0.05) and pathological scores in the bladder and kidneys (*P* < 0.05) and increased the survival rate by 60% (*P* < 0.05). This study evaluated the combination of AZT and antibiotics to treat drug-resistant *K. pneumoniae* infections and found novel drug combinations for the treatment of acute urinary tract infections. These findings suggest that AZT may exert significant anti-resistance activity.

## INTRODUCTION

With antibiotic resistance rapidly spreading, multidrug-resistant (MDR) Gram-negative bacteria (GNB) have become a major public health concern, threatening our ability to effectively treat bacterial infections (1, 2). MDR *Klebsiella pneumoniae* has been listed as a key pathogen by the World Health Organization, and new antibiotics to treat these infections are urgently needed. *K. pneumoniae* is a common pathogen that causes serious human infections, including bacteremia, pneumonia, and meningitis (3, 4). Remarkably, *K. pneumoniae* is adept at acquiring genes, through mutations and/or mobile genetic elements, that encode mechanisms for antibiotic resistance (5). Globally, the prevalence of *K. pneumoniae* strains resistant to nearly all available antibiotics continues to rise (6). Therefore, new antibiotic therapies are urgently required to cope with the resulting public health crises (7).

Pathogens may inevitably become resistant to any agent, and traditional broad-spectrum antibiotics, including quinolones, tetracycline, and furantoin, often fail against clinically isolated strains (8); therefore, new antimicrobial agents are required to overcome antimicrobial resistance (9). New drug development and the repurposing of drugs approved for other diseases are two typical ways of identifying new antimicrobial therapies. Although new drug development has the potential for significant breakthroughs, research and development risks are high; moreover, the rate of evolution of drug-resistant bacteria outpaces current drug development programs (10). In addition, repurposing existing drugs is an innovative and promising option for the rapid development of novel, safe, and effective antimicrobial alternatives (11, 12). Clinically, antibiotics are often used in combination to treat patients with drug-resistant *K. pneumoniae* infections, which can lead to improvements in treatment outcomes. Therefore, combining existing antibiotics with FDA-approved drugs is an efficient strategy for identifying novel antimicrobial therapies.

Zidovudine (AZT) is an effective FDA-approved HIV reverse transcriptase inhibitor. Although AZT has been reported as effective against bacterial infections, its mechanism of action remains uncertain. One theory postulates that AZT may be phosphorylated by thymidine kinase and incorporated into bacterial DNA, acting as a chain terminator (13, 14), similar to its mechanism in HIV. The function of bacterial thymidine kinases, especially those of *Escherichia coli*, support this hypothesis (15). In GNB cells, AZT has been shown to be phosphorylated by thymidine kinases and incorporated into DNA, thereby arresting replication by acting as a DNA-chain terminator (16). Thus, AZT has bactericidal activity against GNB, including *E. coli* and *K. pneumoniae* (17–19), and was shown to be efficacious in a rat pyelonephritis model of systemic *E. coli* infection (20).

Additionally, several *in vitro* and *in vivo* studies have demonstrated synergistic antimicrobial activity against MDR *Enterobacteriales* when AZT is administered in combination with several antibiotics, including colistin, tigecycline, fosfomycin, carbapenems, and trimethoprim (18, 21–25). Given that polymyxins permeabilize the outer membrane of GNB (26), their exposure likely enhances the antimicrobial activity of AZT by increasing its intracellular concentration, thereby maximizing the interaction with intracellular targets (27). Liu et al. concluded that AZT acts synergistically with tigecycline by interfering with bacterial DNA synthesis and inhibiting the activity of the flavin adenine dinucleotide-dependent monooxygenase Tet(X3/X4) (28). However, despite these studies examining lipopeptide antibiotics, most studies on the effects of concomitant AZT and antibiotics have been conducted *in vitro*. Although AZT has been shown to be active against GNB (20, 29), leading to the discovery that AZT is a potent agent capable of sensitizing MDR *K. pneumoniae* to antibiotics, whether the concentrations used clinically are sufficient to treat bacterial infections in humans is unknown. Moreover, the synergy between nitrofuran antibiotics and AZT has not been sufficiently investigated.

Based on previous experiences regarding the pathogenesis and related mechanisms of *K. pneumoniae* (30, 31), we aimed to explore potential drug combinations against MDR *K. pneumoniae*. Thus, the primary objective of this study was to evaluate the *in vitro* and *in vivo* efficacy of AZT in combination with nitrofurantoin (NIT) or omadacycline (OMC) against MDR *K. pneumoniae*.

## MATERIALS AND METHODS

### Bacterial isolates, antimicrobials, and mice

From January 2017 to December 2020, 12 nonrepetitive *K. pneumoniae* strains were isolated from clinical samples of inpatients and outpatients from the Anhui Center for Surveillance of Bacterial Resistance with qualified data. All isolates were stored in Mueller–Hinton broth (MHB; Sigma-Aldrich, St. Louis, MO, USA) with 50% glycerol in cryovials at −80°C and grown on Muller–Hinton agar (MHA; Sigma-Aldrich) at 37°C. *K. pneumoniae* ATCC 43816 was the wild-type standard strain for quality control.

All antibiotics were purchased from Sigma-Aldrich. An FDA-approved drug library was purchased from Selleckchem (Houston, TX, USA). All prepared solutions were stored at −80°C for for ≤1 month.

Wild-type female C57BL/6 mice (8 weeks, 16–20 g) were purchased from the Experimental Animal Center of the Anhui Province (Hefei, China).

### Antimicrobial susceptibility testing

Susceptibility assays were performed using the broth dilution method following CLSI recommendations (32). The density of the isolated colonies was standardized to 0.5 McFarland standard turbidity (~1.5 × 10^8^ CFU/mL) with sterile saline, and adjusted to a bacterial concentration of 1.5 × 10^6^ CFU/mL with MHB. The ﬁnal drug concentrations in the wells ranged from 1–512 mg/L; MHB was used as a negative control, and a drug-free well was set as the growth control. The 96-well plates were subsequently incubated at 37°C for 16–18 h. Minimum inhibitory concentrations (MICs) were defined as the lowest concentration that inhibited visible bacterial growth. Susceptibility was determined using breakpoints from the CLSI guidelines (32). All MICs were tested in quadruplicate.

### Microdilution checkerboard assays

The interaction between two compounds was assessed using microdilution checkerboard assays, as previously reported (33). Fractional inhibitory concentration indices (FICIs) were calculated by first determining the MIC of each drug individually. Following this, the FICI of each drug in combination was determined by using the formula:


$$FICI=\left(\frac{MIC\;of\;drug\;A\;in\;combination}{MIC\text{​}\;of\;drug\;A\text{​}\;alone}\right)+\left(\frac{MIC\;of\;drug\;B\;in\;combination}{MIC\text{​}\;of\;drug\;B\text{​}\;alone}\right)$$

FICI values ≤0.5 indicated synergy (SYN), 0.5–4 indicated indifference (IND), and >4 indicated antagonism (34).

### Growth curve determination

Growth curves were assessed using overnight bacterial cultures, diluted to a density of 10^6^ CFU/mL in MHB. In a 96-well plate, 100 µL of bacterial culture medium containing AZT, NIT, OMC, or AZT + NIT/OMC was cultured at 37°C in an automatic microplate reader (Tecan). OD_600_ readings were recorded every 60 min (35).

### Spot dilution assay

A spot dilution test was performed as described previously (36). Overnight *K. pneumoniae* GN 172867 cultures were diluted to 10^6^ CFU/mL in MHB containing various drugs and added to 96-well plates. Cell viability was monitored by counting CFUs at 0, 12, and 24 h. The chemosensitizing activity of AZT was further validated by spotting 10-µL aliquots of treated and non-treated cultures on MHA plates. The plates were incubated at 37°C overnight before scanning. Histograms were constructed by plotting CFUs from different treatments at each timepoint, using the mean values of three independent experiments.

### Time-dependent killing

Suspensions of *K. pneumoniae* GN 172867 were diluted to 10^6^ CFU/mL in MHB and treated with different drugs at 37°C with continuous shaking (220 rpm). At 0, 4, 8, and 12 h, 10-µL aliquots were removed, centrifuged, resuspended in PBS, and serially diluted. These dilutions were spotted on MHA, and colony counts were determined after overnight incubation at 37°C. CFUs from different treatments were plotted for each timepoints, with three replicates for all experiments (37).

### Determination of biofilm biomass by crystal violet assay

In our study, we employed two distinct assays to evaluate the effects of the tested compounds on biofilm formation and on preformed biofilms. For the assessment of biofilm formation, we used a method that involves the direct exposure of bacterial cultures to the compounds during the initial stages of biofilm development. This approach allows us to observe the impact of the compounds on the early stages of biofilm formation, including bacterial adhesion and microcolony formation. Specifically, bacterial suspensions (200 µL, 10^6^ CFU/mL) were treated with 0.125 mg/L AZT, 4 mg/L NIT, or both, transferred to a 96-well plate, and cultured at 37°C for 36 h. In contrast, the assay for preformed biofilms involves the treatment of established biofilms with the compounds. This method is designed to simulate the conditions where biofilms have already formed and are exposed to treatment, enabling us to assess the compounds’ activity to disrupt or reduce the viability of mature biofilms. Specifically, bacterial suspensions (200 µL, 10^6^ CFU/mL) were transferred to a 96-well plate, cultured at 37°C for 24 h, and the resultant biofilms were treated with 0.125 mg/L AZT, 4 mg/L NIT, or both. The incubation was then continued at 37°C for 12 h. For both methods, the wells were subsequently rinsed with PBS without destroying the established biofilm. After a 10-min methanol fixation, the biofilms were stained with 0.1% crystal violet solution for 10 min, followed by a rinse with water. After drying, the stained biofilms were photographed before adding 33% acetic acid and measuring OD_590_ (38).

Both assays are critical for a comprehensive understanding of the antimicrobial effects of the compounds on biofilms at different stages of development.

### Murine urinary tract infection (UTI) model

C57BL/6 mice were used for a previously described unobstructed ascending UTI model (39). The bacterial inoculum was prepared by overnight incubation of *K. pneumoniae* GN172867 at 37°C in MHB, followed by reinoculation in fresh MHB. The cells were centrifuged at 8,000 rpm for 10 min and diluted in 10 mL of sterile PBS for a final density of 3.0 × 10^7^ CFU/mL.

After anesthetization with isoflurane, a syringe covered with a sterile urinary catheter was inserted into the urethra, and 50 µL of bacterial inoculum or sterile PBS (control group) was deposited into the bladder. After 24 h, mice were treated with AZT (intraperitoneally [i.p.], 10 mg/kg/day), NIT (i.p., 5 mg/kg/day), or both. PBS and DMSO were used as solvents, with <1% DMSO at the final concentration; the control and model group mice were i.p. injected with the same volume of drug-free solvents.

At 48 h post-infection, urine was collected using sterile tubes and centrifuged. Enzyme-linked immunosorbent assays (ELISAs) were used to assess the following urine inflammatory indices: interleukin (IL)-1β, IL-6, and tumor necrosis factor (TNF)-α.

Mice were subsequently euthanized with isoflurane, and the bladder (divided into two) and kidneys were removed for analysis. To determine bacterial density, the bladders/kidneys were homogenized in 1-mL PBS and transferred into a sterile test tube for serial dilution; 100 µL was cultured on broth agar to assess viability after overnight incubation at 37°C. For histological examination, 4-µm sections of formalin-fixed paraffin-embedded tissues were stained with hematoxylin and eosin (HE).

Pathology scoring was based on a previously described method (40). Bladder tissues were evaluated for histopathological characteristics, including epithelial edema, hemorrhage, epithelial thinning, and inflammatory cell infiltration, and scored as follows: 0, normal bladder; 1, minimal damage; 2, mild damage; 3, moderate damage; and 4, severe damage. Kidney tissues were evaluated for histopathological characteristics, including tubular dilatation, tubular cell vacuolization/degeneration and spilling, glomerular atrophy or degeneration, dilatation in the Bowman’s capsule, and separation in the parietal layer of the Bowman’s capsule, and scored as follows (41): 0, normal (no damage); 1, minimal damage (<25%); 2, mild damage (25–50%); 3, moderate damage (50–75%); and 4, severe damage (>75%).

### Murine lung infection model

We established lung infection models based on our previous studies (42). C57BL/6 mice were anesthetized with isoflurane and inoculated intranasally with 50 µL of sterile PBS (control group) or 1.5 × 10^8^ CFU of *K. pneumoniae* GN172867 in 50 µL of sterile PBS. After 3 h, they were treated with AZT (i.p., 10 mg/kg/day), OMC (i.p., 4 mg/kg/day), or both. PBS and DMSO were used as solvents, with <1% DMSO at the final concentration; control and model group mice were i.p. injected with the same volume of drug-free solvents.

After 24 h of infection, the mice were euthanized, and the lungs were dissected. For lung colony counts, lung tissue was homogenized in 1-mL PBS and transferred into a sterile test tube for serial dilution; 100 µL was cultured onto broth agar to assess viability after overnight incubation at 37°C. The data were expressed as means ± standard deviation log_10_ CFU/mouse.

For pathological assessment, the whole left lung was excised aseptically, and the pathological diagnosis was pneumonia. For histological evaluation, 4-µm formalin-fixed paraffin-embedded sections were stained with HE. Pathology scoring was based on a previously described method (42) where the total lung inflammation score was the sum of the scores for each parameter, with a maximum of 24.

Whole blood was obtained using the mouse eyeball enucleation method 24 h after inoculation, and serum was collected after centrifugation. ELISA was used to measure the inflammatory indices IL-1β, IL-6, and TNF-α.

For survival curve assessments, the mice were monitored 4–5 times daily and euthanized when they exhibited ≥2 of the following signs of systemic pneumococcal infection: reduced movement, hunched posture, piloerection, shivering, dyspnea, or circling.

### Statistical analysis

Data were presented as the means of three independent experiments, and error bars represent the standard errors of the means. All statistical analyses were performed using unpaired Student *t*-tests for two groups and one- or two-way analyses of variance for multiple groups, with all data points showing a normal distribution. Survival curves were plotted using the Kaplan–Meier method and compared with log-rank tests. Statistical significance is indicated as follows: ns, no significance; *, *P* < 0.05; **, *P* < 0.01; ***, *P* < 0.001; and ****, *P* < 0.0001. All graphs were generated using GraphPad Prism 8.0 (GraphPad Inc., San Diego, CA, USA), FlowJo version 10.4 (Ashland, OR, USA), and Adobe Illustrator CC 2021 (Adobe Systems Inc., San Jose, CA, USA).

## RESULTS AND DISCUSSION

### Antimicrobial activity of multiple antibiotics combined with AZT

Susceptibility testing was performed on 12 clinical isolates of MDR *K. pneumoniae* (Table 1); the MDR strain GN 172867 was randomly selected as the test strain.

**TABLE 1:** Antimicrobial susceptibility for clinically isolated *K. pneumoniae* strains used in this study^*a*^^, *b*^

	Quinolones		Nitrofurans	Tetracyclines			Penicillins	Cephalosporin alkene	Cephalosporin alkene		Monocyclic lactam
Strains	CIP	LVX	NIT	TCY	DOX	OMC	AMP	PIP	CRO	CXM	ATM
GN 170843	1024 (R)	128 (R)	128 (R)	>128 (R)	32 (R)	16 (R)	>256 (R)	>1024 (R)	>32 (R)	>256 (R)	64 (R)
GN 172055	256 (R)	128 (R)	512 (R)	128 (R)	32 (R)	16 (R)	>256 (R)	512 (R)	>32 (R)	>256 (R)	16 (R)
**GN 172867**	**256** (R)	**128** (R)	**256** (R)	**>128** (R)	**32** (R)	8 (I)	**>256** (R)	**>1024** (R)	**>32** (R)	**>256** (R)	**64** (R)
GN 180762	256 (R)	128 (R)	256 (R)	64 (R)	8 (I)	16 (R)	>256 (R)	>1024 (R)	>32 (R)	>256 (R)	128 (R)
GN 183491	256 (R)	256 (R)	256 (R)	>128 (R)	128 (R)	32 (R)	>256 (R)	512 (R)	32 (R)	256 (R)	4 (S)
GN 191034	256 (R)	128 (R)	128 (R)	>128 (R)	64 (R)	32 (R)	>256 (R)	1024 (R)	>32 (R)	>256 (R)	64 (R)
GN 192105	256 (R)	256 (R)	128 (R)	>128 (R)	32 (R)	16 (R)	>256 (R)	>1024 (R)	>32 (R)	>256 (R)	>128 (R)
GN 192428	256 (R)	128 (R)	128 (R)	>128 (R)	64 (R)	16 (R)	>256 (R)	>1024 (R)	>32 (R)	>256 (R)	128 (R)
GN 200906	256 (R)	256 (R)	256 (R)	>128 (R)	64 (R)	32 (R)	>256 (R)	512 (R)	>32 (R)	>256 (R)	64 (R)
GN 201292	256 (R)	>256 (R)	256 (R)	>128 (R)	128 (R)	64 (R)	>256 (R)	1024 (R)	>32 (R)	>256 (R)	128 (R)
GN 203463	256 (R)	128 (R)	128 (R)	32 (R)	64 (R)	8 (I)	>256 (R)	>1024 (R)	>32 (R)	>256 (R)	128 (R)
GN 203937	256 (R)	128 (R)	128 (R)	>128 (R)	128 (R)	2 (S)	>256 (R)	>1024 (R)	>32 (R)	>256 (R)	64 (R)
ATCC 43816	0.06 (S)	0.13 (S)	64 (I)	4 (S)	2 (S)	1 (S)	8 (S)	≤0.06 (S)	8 (S)	≤0.25 (S)	≤0.25 (S)

	Carbapenems		Lipopeptides	Aminoglycosides		Folic acid metabolicpathway inhibitors		Styrene acrylic alcohols	Fosfomycin class
Strains	IMP	MEM	COL	GEN	AMK	SXT	TMP	CHL	FOS
GN 170843	1 (S)	≤0.06 (S)	1 (S)	>128 (R)	>512 (R)	>32 (R)	>128 (R)	>256 (R)	>2048 (R)
GN 172055	1 (S)	≤0.06 (S)	1 (S)	2 (S)	2 (S)	>32 (R)	>128 (R)	>256 (R)	64 (S)
**GN 172867**	**1** (S)	**≤0.06** (S)	**1** (S)	**64** (R)	**2** (S)	**>32** (R)	**>128** (R)	**>256** (R)	**2048** (R)
GN 180762	1 (S)	≤0.06 (S)	1 (S)	>128 (R)	>512 (R)	>32 (R)	>128 (R)	64 (R)	256 (R)
GN 183491	0.5 (S)	≤0.06 (S)	1 (S)	1 (S)	2 (S)	>32 (R)	>128 (R)	64 (R)	512 (R)
GN 191034	2 (I)	≤0.06 (S)	>32 (S)	128 (R)	>512 (R)	>32 (R)	>128 (R)	>256 (R)	64 (S)
GN 192105	>32 (R)	16 (R)	1 (S)	>128 (R)	>512 (R)	>32 (R)	>128 (R)	64 (R)	32 (S)
GN 192428	2 (I)	≤0.13 (S)	1 (S)	128 (R)	4 (S)	>32 (R)	>128 (R)	>256 (R)	256 (R)
GN 200906	0.5 (S)	0.13 (S)	0.5 (S)	64 (R)	1 (S)	>32 (R)	>128 (R)	>256 (R)	32 (S)
GN 201292	4 (R)	≤0.06 (S)	1 (S)	32 (R)	4 (S)	>32 (R)	>128 (R)	>256 (R)	64 (S)
GN 203463	2 (I)	0.06 (S)	0.5 (S)	8 (I)	4 (S)	>32 (R)	>128 (R)	>256 (R)	128 (I)
GN 203937	0.5 (S)	≤0.06 (S)	1 (S)	64 (R)	1 (S)	>32 (R)	>128 (R)	>256 (R)	128 (I)
ATCC 43816	0.5 (S)	≤0.06 (S)	0.5 (S)	0.5 (S)	≤1 (S)	0.25 (S)	2 (S)	8 (S)	128 (I)

^*a*^ MIC, minimum inhibitory concentration; R, resistant; I, intermediate; S, sensitive; CIP, ciprofloxacin (the range of ciprofloxacin concentrations used in the MIC test was 0.016–8 mg/L); LVX, levofloxacin (0.0313–16 mg/L); NIT, nitrofurantoin (2–1,024 mg/L); TCY, tetracycline (0.5–128 mg/L); DOX, doxycycline (0.25–128 mg/L); AMP, ampicillin (0.5–256 mg/L); PIP, piperacillin (1–1,024 mg/L); CRO, ceftriaxone (0.125–32 mg/L); CXM, cefuroxime (0.5–256 mg/L); ATM, aztreonam (0.25–128 mg/L); IMP, imipenem (0.125–32 mg/L); MEM, meropenem (0.125–32 mg/L); COL, colistin (0.125–16 mg/L); GEN, gentamicin (0.25–128 mg/L); AMK, amikacin (0.125–16 mg/L); SXT, trimethoprim-sulfamethoxazole (0.125–32 mg/L); TMP, trimethoprim (0.5–128 mg/L); CHL, chloramphenicol (0.5–256 mg/L); FOS, fosfomycin (4–2,048 mg/L).

^*b*^ The bolded MIC values indicate the minimum concentration of the antibiotic required to inhibit the growth of the bacterial strain.

AZT exhibits potent antibacterial and chemosensitizing activities. Several *in vitro* and *in vivo* studies using MDR *Enterobacteriales* have demonstrated the synergistic antimicrobial activity of AZT in combination with several antibiotics, including colistin, tigecycline, fosfomycin, carbapenems, and trimethoprim (21); however, the mechanisms of action remain unknown. Therefore, further in-depth studies examining the broad-spectrum reversal activity of AZT against resistant bacteria are required. The results in Table 2 show that AZT enhanced the antibacterial activity of five antibiotics, including NIT, OMC, levofloxacin, ciprofloxacin, and tetracycline, indicating that AZT has the potential to reverse GN 172867 drug resistance.

**TABLE 2:** Effect of zidovudine on the antibacterial activity of five antibiotics against *K. pneumoniae* GN 172867^*a*^

		MIC (mg/L)			
Compound A	Compound B	Alone	Combined	FICI	Mode
Zidovudine		4	0.25	0.31	SYN
	Levofloxacin	128	32		
Zidovudine		4	0.13	0.28	SYN
	Ciprofloxacin	256	64		
Zidovudine		4	0.25	0.19	SYN
	Nitrofurantoin	256	32		
Zidovudine		4	0.25	0.31	SYN
	Tetracycline	256	64		
Zidovudine		4	1	0.50	SYN
	Omadacycline	8	2		

^*a*^ SYN, synergy; IND, indifference; FICI, fractional inhibitory concentration index.

Notably, AZT showed the best synergy with NIT, although the new tetracycline drug OMC also demonstrated potential activity against MDR *K. pneumoniae* in combination with AZT. Clinically, NIT is widely used to treat urinary tract infections. OMC is a C9-aminomethyl modification of minocycline, which allows OMC to overcome bacterial resistance and expand its antibacterial spectrum, with improved pharmacokinetic properties (43, 44). Furthermore, OMC is available in both oral and intravenous formulations (45). To date, OMC has been indicated for community-acquired bacterial pneumonia (46). To the best of our knowledge, the synergy between NIT/OMC and AZT has not previously been reported. Therefore, we focused on AZT + NIT or OMC to characterize synergistic activity.

**Fig 1:**
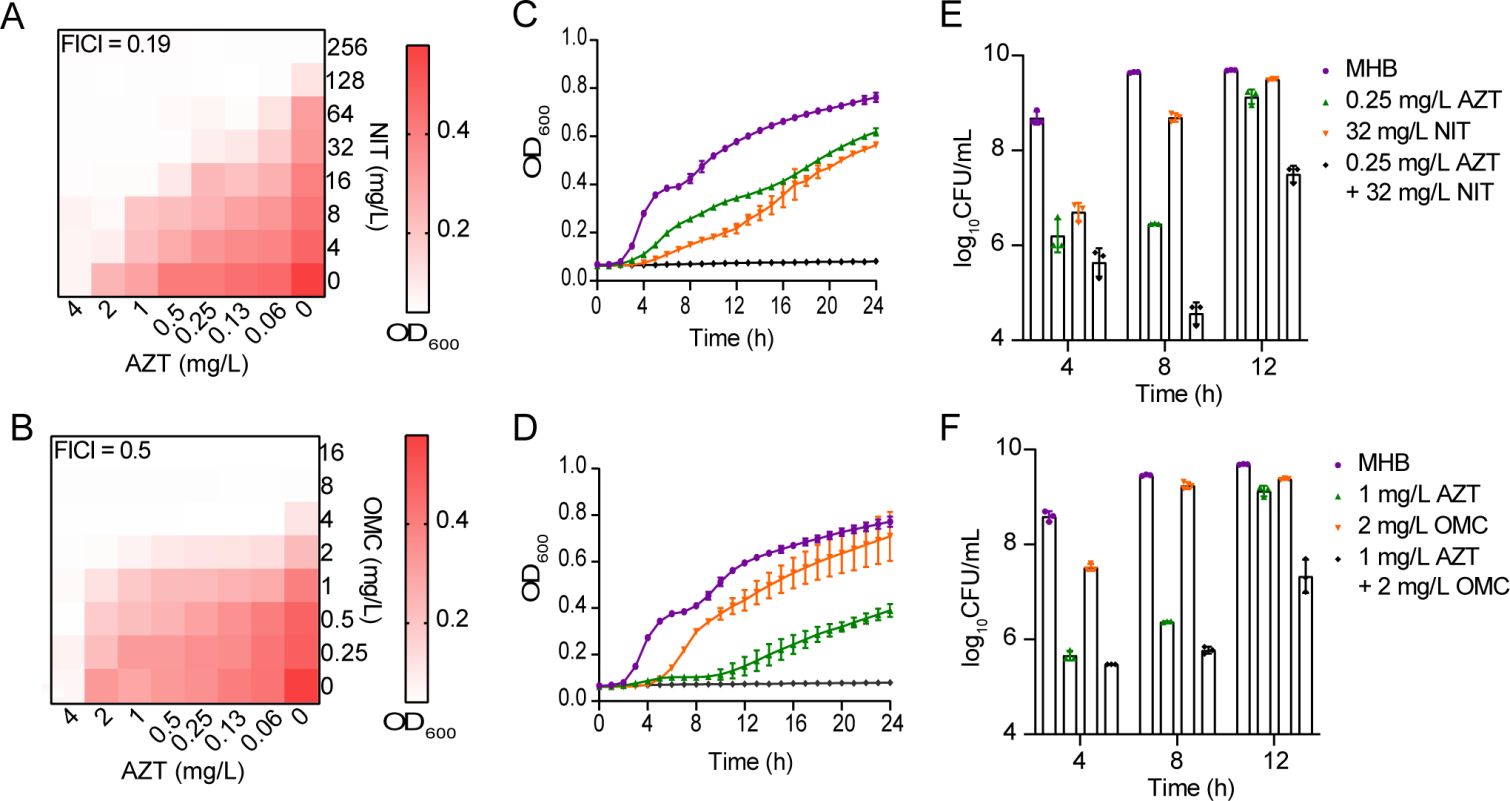
AZT drastically potentiates NIT and OMC activities against *K. pneumoniae* GN 172867. (**A and B**) Checkerboard broth microdilution assays between AZT and NIT/OMC against GN 172867. Dark red regions represent higher bacterial cell densities and lower inhibition rate of combinational treatment. Synergy is defined as an FIC index of ≤0.5. (**C**) Growth curve of GN 172867 under 0.25 mg/L AZT, 32 mg/L NIT, or a combination of both (AZT + NIT). The drugs were assessed against GN 172867 for 24 h at 37°C; planktonic cell growth was detected by OD_600_ analysis. (**D**) Growth curve of GN 172867 under 1 mg/L AZT, 2 mg/L OMC, or a combination of both (AZT + OMC). (**E**) Time-dependent killing of pathogens by the combination of AZT and NIT. GN 172867 was grown to late exponential phases in MHB broth, then treated with MHB, 0.25 mg/L AZT or 32 mg/L NIT alone or in combination (AZT + NIT). The bacterial CFUs/mL at different timepoints during 12 h were determined. (**F**) Time-dependent killing of pathogens by the combination of AZT and OMC. GN 172867 was grown to late exponential phases in MHB broth, then treated with MHB, 1 mg/L AZT or 2 mg/L OMC alone or in combination (AZT + OMC). The bacterial CFUs/mL at different time points during 12 h were determined. All experiments were performed thrice, and the mean ± SD is shown.

**Fig 2:**
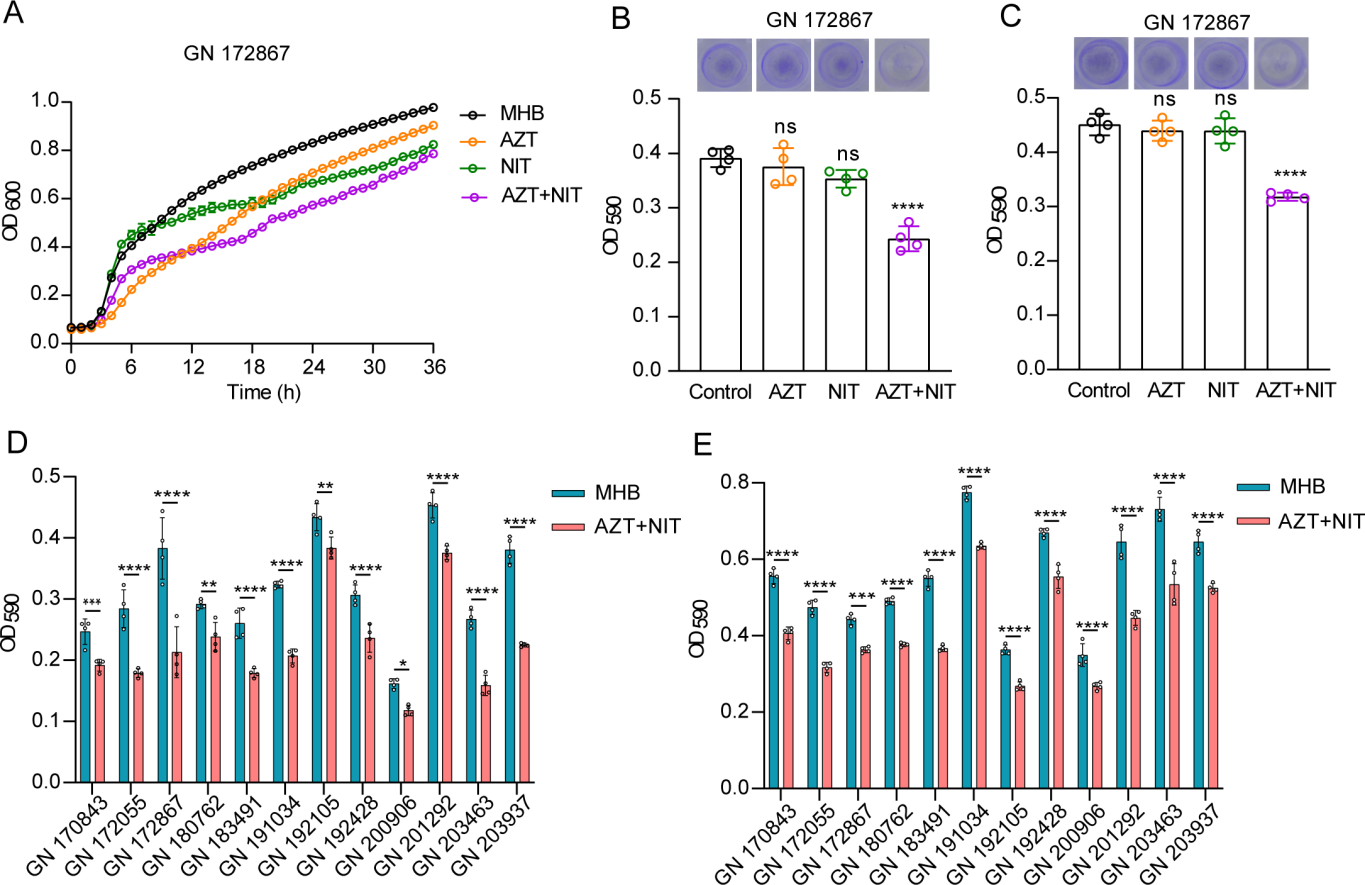
Effects of AZT and NIT on *K. pneumoniae* biofilms. (**A**) GN 172867 was treated with 0.125 mg/L AZT, 4 mg/L NIT, 0.125 mg/L AZT + 4 mg/L NIT, or not; planktonic cell growth was detected by OD_600_ analysis. (**B**) AZT combined with NIT inhibited the formation of GN 172867 biofilm. GN 172867 was treated with 0.125 mg/L AZT or 4 mg/L NIT or a combination of both for 36 h; 0.1% crystal violet-stained biofilms were detected by OD_590_ analysis. (**C**) AZT combined with NIT inhibited biofilm formation in 12 strains of *K. pneumoniae*. (**D**) Clearance of mature biofilm of GN 172867 by a combination of AZT and NIT. GN 172867 was first cultured for 24 h without the addition of drugs, and then treated with 0.125 mg/L AZT or 4 mg/L NIT or a combination of both for 12 h; 0.1% crystal violet-stained biofilms were detected by OD_590_ analysis. (**E**) Effect of AZT combined with NIT on the clearance of mature biofilms of 12 strains. All data are presented as mean ± standard deviation (*n* = 4 biological replicates). Error bars represent standard errors of the means. Statistically significant differences are indicated as follows: ns, no significance, *P < 0.05, **P < 0.01, ***P < 0.001, ****P < 0.0001.

The microdilution checkerboard showed that AZT exhibited synergistic effects with NIT (FICI = 0.31) and OMC (FICI = 0.50) against *K. pneumoniae* GN 172867, suggesting that AZT enhanced their growth inhibitory effects (Fig. 1A and B). Similarly, the bacterial growth curves demonstrated that the combinations AZT + NIT and AZT + OMC effectively inhibited the growth of *K. pneumoniae* GN 172867 (Fig. 1C and D). The spot dilution assay showed that compared with the no-drug group, bacterial growth was almost unaffected by AZT, NIT, or OMC alone (10^9^ CFU/mL), whereas AZT + NIT and AZT + OMC had a synergistic bactericidal effect (Fig. S1A and B). In addition, time-dependent killing experiments showed that the colony count decreased by ≥2 log10, 8 h after AZT + NIT or AZT + OMC treatment compared with the most effective single-drug group (Fig. 1E and 2F).

### Broad-spectrum activity of AZT+NIT and AZT+OMC

We used the checkerboard method to assess the antimicrobial activity and potential synergistic effects of the novel drug combinations AZT + NIT and AZT + OMC against 11 other clinically isolated MDR *K. pneumoniae* strains (Table 3). According to the CLSI guidelines (47), an MIC ≥128 mg/L of NIT against *K. pneumoniae* was considered resistant, 32–128 mg/L, intermediate, and ≤32 mg/L, sensitive; for OMC, an MIC ≥16 mg/L was considered resistant, 4–16 mg/L, intermediate, and ≤4 mg/L, sensitive. All 12 clinical strains, including GN 172867, were resistant to NIT (MIC = 128–512 mg/L), whereas for OMC, nine strains were resistant (MIC = 16–64 mg/L), one was sensitive (MIC = 2 mg/L), and two were intermediate (MIC = 8 mg/L). In addition, the AZT MICs against the 12 strains ranged from 2 to 32 mg/L. The combination AZT + NIT showed a synergistic effect on all 12 strains (100%); with an AZT concentration between 0.25 and 2 mg/L, the NIT MICs decreased 4- to 16-fold.

**TABLE 3:** Effect of zidovudine on the antibacterial activity of omadacycline and nitrofurantoin against 12 clinical *K. pneumoniae* isolates^*a*^

	Data for NIT-AZT:				Data for OMC-AZT:			
	MIC (mg/L)				MIC (mg/L)			
Bacterial isolate	Alone	Combined	FICI	Mode	Alone	Combined	FICI	Mode
GN 170843	128/8	16/1	0.25	SYN	16/8	8/0.13	0.52	IND
GN 172055	512/8	32/2	0.32	SYN	16/8	2/1	0.25	SYN
**GN 172867**	256/4	32/0.25	0.19	SYN	8/4	2/1	0.5	SYN
GN 180762	256/4	32/0.25	0.19	SYN	16/4	2/1	0.38	SYN
GN 183491	256/4	32/0.5	0.25	SYN	32/4	16/0.13	0.53	IND
GN 191034	128/2	16/0.25	0.25	SYN	32/2	16/0.13	0.57	IND
GN 192105	128/4	16/0.25	0.19	SYN	16/4	8/1	0.75	IND
GN 192428	128/4	16/0.25	0.19	SYN	16/4	8/0.5	0.63	IND
GN 200906	256/4	32/0.5	0.25	SYN	32/4	16/0.25	0.57	IND
GN 201292	256/2	32/0.5	0.38	SYN	64/2	16/1	1	IND
GN 203463	128/2	16/0.25	0.25	SYN	8/2	4/1	1	IND
GN 203937	128/32	32/0.25	0.26	SYN	2/32	0.5/32	1.25	IND

^*a*^ SYN, synergy; IND, indifference; AZT, zidovudine; NIT, nitrofurantoin; OMC, omadacycline; FICI, fractional inhibitory concentration index.

Four strains (including carbapenem-resistant *K. pneumoniae* GN 192105) showed the best synergistic effects (FICI = 0.19); the MIC of AZT alone was 4 mg/L, and the range of NIT alone was 128–256 mg/L. The strain with the worst synergistic effect was GN201292; the MIC was 256 mg/L with NIT alone and 32 mg/L when combined with 0.5 mg/L AZT (FICI = 0.38), whereas the MIC was 2 mg/L with AZT alone.

AZT + OMC showed synergistic effects in only three strains (GN 172055, FICI = 0.25; GN 172867, FICI = 0.5; and GN 180762, FICI = 0.38), none of which were OMC-sensitive strains; the MIC value of OMC was reduced 4- to 8-fold when combined with 1 mg/L AZT. However, no synergistic effects were observed in the other nine (75%) OMC-non-sensitive strains. The FICIs of the 12 strains showed no obvious relationship between AZT and reversal of OMC resistance.

### Effects of AZT and NIT on *K*. *pneumoniae* biofilms

Previous reports have demonstrated that AZT inhibits biofilm formation (11, 48, 49); therefore, we performed crystal violet staining of *K. pneumoniae* GN 172867 biofilms and assessed the relative biomass. First, different concentrations of AZT or NIT were examined, and 0.125 mg/L AZT and 4 mg/L NIT did not significantly inhibit bacterial growth (Fig. 2A; Fig. S2). The concentrations of each drug selected for our experiments were chosen based on growth curve studies that identified the concentrations that do not exhibit growth inhibition as a single agent. This approach was taken to ensure that any observed inhibitory effects in combination treatments could be attributed to a synergistic interaction rather than the inhibitory action of the individual drugs at higher concentrations. By focusing on non-inhibitory concentrations, we aimed to delineate the potential for these drugs to work in concert to enhance their antimicrobial effects. Next, GN 172867 was treated with or without 0.125 mg/L AZT, 4 mg/L NIT, or both for 36 h, and biofilms were detected at OD_590_ (Fig. 2B); AZT +NIT caused a significant decrease in biofilm formation compared with control and AZT or NIT monotherapy (*P* < 0.0001). Moreover, the combination AZT + NIT promoted the clearance of preformed *K. pneumoniae* biofilms compared with monotherapy (Fig. 2C). In addition, AZT + NIT inhibited biofilm formation (Fig. 2D) and dispersed mature biofilms (Fig. 2E) in the 12 strains of *K. pneumoniae*. Taken together, these data suggest that AZT + NIT had both antibiofilm activity and eradicated established biofilms *in vitro* without affecting bacterial growth.

### Antimicrobial efficacy of AZT+NIT in a murine UTI model

**Fig 3:**
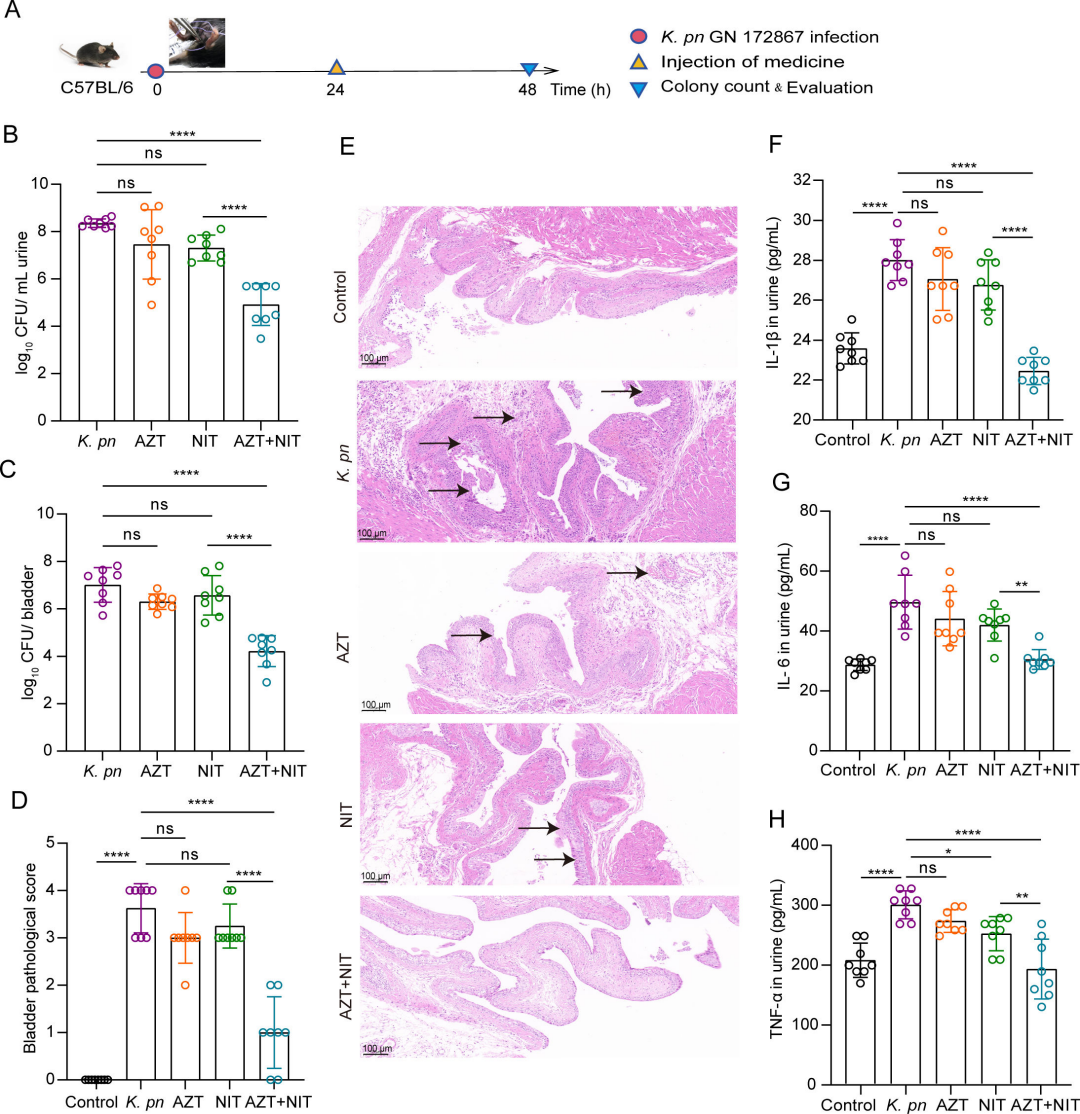
Effect of AZT and NIT on *K. pneumoniae* GN 172867-induced in murine UIT. (**A**) Schematic diagram of UIT and treatment process. C57BL/6 mice were infected with 10^7^ CFU of *K. pneumoniae* via urethra. Then, 24 h after infection, the mice were treated with 10 mg/kg AZT (AZT group), 5 mg/kg NIT (NIT group), or a combination of AZT and NIT (AZT + NIT group) by intraperitoneal injection. The PBS group was used as a control. Another group (no infection group) that was injected with 50 µL of sterile PBS instead of *K. pneumoniae* was used as a negative control. Treatment was administered once. The group size was 8 mice. (**B**) After 48 h of infection with *K. pneumoniae*, the urine bacterial load was measured in the treated and untreated groups. (**C**) After 48 h of infection with *K. pneumoniae*, the bladder bacterial load was measured in the treated and untreated groups. (**D and E**) Representative bladder tissue slices of untreated and treated groups after *K. pneumoniae* infection; total bladder histopathology scores. The arrows indicate histopathological characteristics, including epithelial edema, hemorrhage, epithelial thinning, and inflammatory cell infiltration. HE staining scale bars = 100 µm (10×). (**F, G and H**) Urine inflammatory index of urinary tract infection in mice infected with *K. pneumoniae*. The levels of inflammatory urine markers (IL-1β, IL-6, and TNF-α) of urinary tract infection. All data are expressed as mean ± standard deviation, ns, no significance, *P < 0.05, **P < 0.01, ***P < 0.001, ****P < 0.0001.

After *E. coli*, *K. pneumoniae* is the second most common pathogen that causes UTIs (50, 51). Nitrofurans, such as NIT, are the most commonly used drugs for the clinical treatment of recurrent UITs; however, the emergence of MDR strains has rendered these agents ineffective at normal doses (52). Based on our *in vitro* experiments, we further evaluated the synergistic antibacterial effect of AZT + NIT in a GN 172867-induced murine UTI model, established according to our previous study (Fig. 3A).

Compared with the model group, the groups treated with AZT or NIT monotherapy did not exhibit significant changes in urine, bladder, and kidney tissue colony counts (*P* > 0.05). In contrast, the AZT + NIT treatment group had significantly reduced colony counts in urine (Fig. 3B), bladder (Fig. 3C), and kidney (Fig. S3A) tissues compared with the model, AZT monotherapy, and NIT monotherapy groups (*P* < 0.0001, *P* < 0.0001, and *P* < 0.05, respectively).

If UTIs are not promptly controlled, ascending pathogenic infections often cause nephritis (53). Thus, we further quantified pathological sections of the bladder and kidney tissues after *K. pneumoniae* infection. The model group displayed histological evidence of severe acute cystitis, as demonstrated by enhanced bladder epithelial edema, hemorrhage, epithelial thinning, and inflammatory cell infiltration (Fig. 3D and E). Compared with the model group, the monotherapy groups showed no significant differences in bladder tissue pathology (*P* > 0.05). In contrast, the AZT + NIT group had significantly reduced bladder epithelial edema and inflammatory cell infiltration compared with the other groups, and the bladder pathological score was statistically significant (*P* < 0.0001). Furthermore, the model group showed histological evidence of tubular dilatation, tubular cell vacuolization/degeneration and spilling, glomerular atrophy/degeneration, dilatation in the Bowman’s capsule, and separation in the parietal layer of the Bowman’s capsule (Fig. S3B and C). The monotherapy groups exhibited no significant differences in renal histopathology compared with the model group (*P* > 0.05). In contrast, the AZT + NIT group had significantly less renal tubule dilatation and glomerular atrophy/degeneration than the other groups, and the pathological score was statistically significant (*P* < 0.0001). These results indicated that GN 172867-induced UTIs could be alleviated by AZT + NIT treatment.

TNF-α, IL-1β, and IL-6 are key pro-inflammatory cytokines involved in a variety of *in vivo* autoimmune inflammatory responses. To explore the infectious status of mice with acute UTIs, we measured the urinary levels of these three inflammatory cytokines. The index values of these factors in the model group were significantly higher than those in the control group (*P* < 0.0001). The index values in the AZT + NIT group were not significantly different from those in the control group (*P* > 0.05). In contrast, the index values were significantly lower in the combination group than in the model and monotherapy groups (*P* < 0.0001) (Fig. 3F through H).

### Antimicrobial efficacy of AZT + OMC in a murine lung infection model

**Fig 4:**
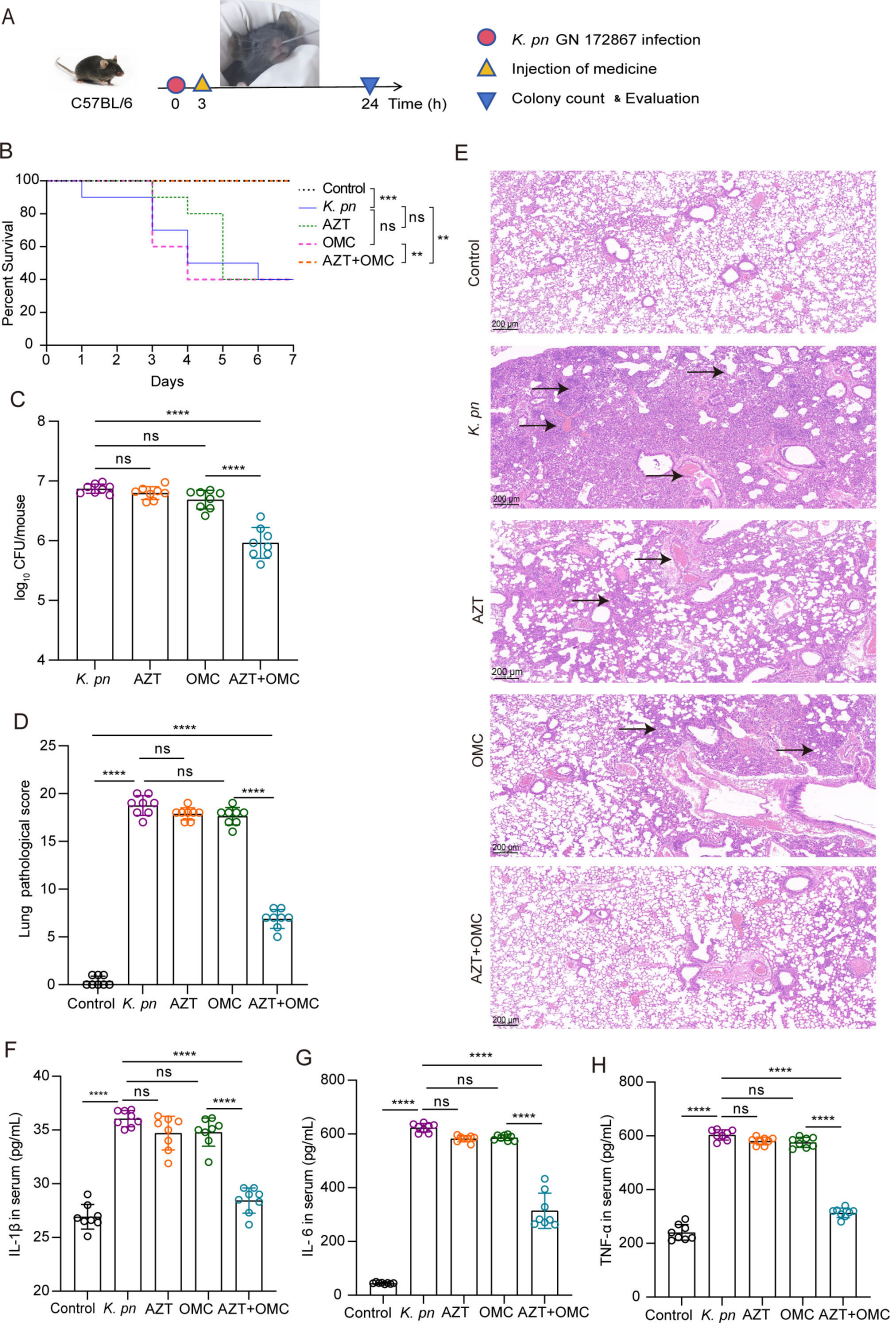
Effect of AZT and OMC on *K. pneumoniae* GN 172867-induced lung infection. (**A**) Schematic diagram of lung infection and treatment process. C57BL/6 mice were infected with 10^8^ CFU of *K. pneumoniae* via the intranasal route. Then, 3 h after infection, the mice were treated with 10 mg/kg AZT (AZT group), 4 mg/kg OMC (OMC group), or a combination of AZT and OMC (AZT + OMC group) by intraperitoneal injection. The PBS group was used as a control. Another group (no infection group) that was injected with 50 µL of sterile PBS instead of *K. pneumoniae* was used as a negative control. Treatment was administered once. (**B**) Kaplan–Meier survival plot of treated and untreated mice after *K. pneumoniae* infection. (**C**) After 24 h of infection with *K. pneumoniae*, the pulmonary bacterial load was measured in the treated and untreated groups. (**D and E**) Representative lung tissue slices of untreated and treated groups after *K. pneumoniae* infection; total lung histopathology scores. The arrows in the figure indicate various pulmonary pathological changes, including bronchitis, interstitial inflammation, edema, endothelitis, and thrombosis. HE staining scale bars = 200 µm (5×). (F, G, and H) Serum inflammatory index of lung infection in mice infected with *K. pneumoniae*. The levels of inflammatory serum markers (IL-1β, IL-6, and TNF-α) of lung infection. Data are from three independent experiments. Statistically significant differences are indicated as follows: ns, no significance, *P < 0.05, **P < 0.01, ***P < 0.001, ****P < 0.0001.

OMC is a newer tetracycline approved for the treatment of bacterial pneumonia. To establish a murine pulmonary infection model, the mice were intranasally inoculated with *K. pneumoniae* GN 172867 at different concentrations. Based on lung tissue colony counts and survival curves, 50 µL of a 3 × 10^9^ CFU/mL bacterial solution was selected as the intranasal inoculation dose, and the model was used to assess the antibacterial effects of AZT + OMC *in vivo* (Fig. 4A; Fig. S4). Remarkably, AZT + OMC significantly improved the survival rate of mice with lung infections (Fig. 4B). Compared with the model group, the AZT and OMC monotherapy groups showed no significant changes in lung tissue colony counts (*P* > 0.05). In contrast, the counts in the AZT + OMC group were significantly lower than those in the model and monotherapy groups (*P* < 0.01) (Fig. 4C).

We further quantified lung pathological sections after GN 172867 infection. The model group showed histological evidence of severe pneumonia, as demonstrated by enhanced alveolar wall destruction, interstitial inflammation, endothelialitis, and edema. The monotherapy groups featured similar lung tissue pathology, with no significant differences compared with the model group (*P* > 0.05). In contrast, the combination group had fewer inflammatory cells and less alveolar wall destruction than the model and monotherapy groups, and the lung pathological score was statistically significant (*P* < 0.0001) (Fig. 4D and E). These results indicated that *K. pneumoniae*-induced lung inflammation can be alleviated by AZT + OMC treatment.

The index values of the serum inflammatory factors (IL-1β, IL-6, and TNF-α) in the model group were significantly higher than those in the control group (*P* < 0.0001). No significant differences in these factors were noted between the AZT and OMC monotherapy groups and the model group (*P* > 0.05), whereas the combination group had significantly lower index values than the model and single-drug groups (*P* < 0.0001) (Fig. 4F through H).

Of note, only female mice were included in these experiments, which limits our ability to understand potential sex-specific differences in the UTI and lung infection models; these differences are conceivable given the known influences of sex hormones. This limitation may also impact the translation of our findings to clinical applications. Both sexes should be included in future studies to provide a more comprehensive understanding of sex differences in the context of the UTI and lung infection models.

### Conclusion

In conclusion, *in vitro* and *in vivo* studies have demonstrated that drug repurposing is an effective method of drug discovery that may solve the current global crisis of antibiotic resistance. The antibacterial activity of AZT has been previously described in mice (20) and has been suspected *in vivo* in humans (16). Moreover, reviving outdated antibiotics may be useful to combat MDR bacteria. In this study, we confirmed that AZT could reverse MDR *K. pneumoniae* GN 172867 drug resistance. These findings suggest that AZT is a potent broad-spectrum antibiotic sensitizer. Importantly, our *in vivo* experiments demonstrated the effectiveness of AZT + NIT and AZT + OMC in terms of their survival benefits, histopathology, and bactericidal effects. Furthermore, the effective concentration of AZT in combination with NIT/OMC should be easily achieved at safe dosages. In addition, AZT + NIT inhibited *K. pneumoniae* biofilm formation and cleared mature biofilms.

In summary, this study identified that the combinations AZT + NIT and AZT + OMC may be effective alternative therapies to treat MDR *K. pneumoniae* infections, which merits further investigation. We believe that AZT is a molecule with unexpressed clinical potential against MDR bacterial infections, especially as part of combination therapy. This early groundwork lays the foundation for further validation in clinical trials, enabling the translation of combination therapy into clinical benefits for patients. However, the optimal dosage, frequency, and synergistic mechanisms require further analysis to achieve better clinical outcomes.

## Data Availability

The data sets used and analyzed during the current study are available from the corresponding author upon reasonable request.

## SUPPLEMENTAL MATERIAL

Supplemental figures aac.00344-24-s0001.docx Figures S1 to S4.

ASM does not own the copyrights to Supplemental Material that may be linked to, or accessed through, an article. The authors have granted ASM a non-exclusive, world-wide license to publish the Supplemental Material files. Please contact the corresponding author directly for reuse.
