# Supplementary material for: Zidovudine in synergistic combination with nitrofurantoin or omadacycline: in vitro and in murine urinary tract or lung infection evaluation against multidrug-resistant Klebsiella pneumoniae
Source: Antimicrob Agents Chemother. 2024 Aug 28;68(10):e00344-24. doi: 10.1128/aac.00344-24 (PMC11459972; doi:10.1128/aac.00344-24)
Supplement: Supplemental figures — Figures S1 to S4. [file aac.00344-24-s0001.docx]

ORIGINAL RESEARCH

Ping Tian et al

**Zidovudine in synergistic combination with nitrofurantoin or omadacycline: *in vitro* and in murine urinary tract or lung infection evaluation against multidrug-resistant** ***Klebsiella pneumoniae***

**Ping Tian^1,2,†^,** **Qing-Qing Li^1,2,†^,** **Ming-Juan Guo^3^, Yun-Zhu Zhu^1,2^, Rong-Qing Zhu^1,2^, Ya-Qin Guo^1,2^, Yi Yang^1,2^, Yan-Yan Liu^1,2^, Liang Yu^1,2*^, Ya-Sheng Li^1,2,*^, and Jia-Bin Li^1,2,*^**

^1^Department of Infectious Diseases & Anhui Center for Surveillance of Bacterial Resistance, The First Affiliated Hospital of Anhui Medical University, Hefei, 230022, China;

^2^ Anhui Province Key Laboratory of Infectious Diseases & Institute of Bacterial Resistance, Anhui Medical University, Hefei, 230022, China;

^3^Department of Hepatology, The First Affiliated Hospital of Jilin University, Changchun, 130021, China

*Correspondence: [yuliang02@ahmu.edu.cn](mailto:lijiabin@ahmu.edu.cn) & liyasheng@ahmu.edu.cn (Y.L.)

&[lijiabin@ahmu.edu.cn](mailto:lijiabin@ahmu.edu.cn) (J.L.)

†The authors contributed equally to this study.

**Supplementary Figure Legends**

**Figure S1.** AZT combined with NIT or OMC significantly inhibited the growth of GN 172867. (A) Spot dilution assay. After 0, 12 and 24 h incubation at 37℃, serial 10-fold dilutions of GN 172867 containing MHB, 0.25 mg/L AZT or 32 mg/L NIT alone or in combination (AZT+NIT) were spotted onto MHA plates. (B) Spot dilution assay. After 0, 12 and 24 h incubation at 37℃, serial 10-fold dilutions of GN 172867 containing MHB, 1 mg/L AZT or 2 mg/L OMC alone or in combination (AZT+OMC) were spotted onto MHA plates. The plates were incubated overnight and then the colony counts were performed.. All experiments were performed three times, and the mean ± SD is shown.

**Figure S2.** Twelve clinical MDR *K. pneumoniae* strains were treated with 0.125 mg/L AZT, 4 mg/L NIT, 0.125 mg/L AZT+4 mg/L NIT, or not for 36 h; planktonic cell growth was detected by OD_600_ analysis. Data are from three independent experiments.

**Figure S3.** Effects of AZT and NIT on kidney in mice with urinary tract infection caused by *K. pneumoniae* GN 172867. (A) After 24 h of infection with K. pneumoniae, the kidney bacterial load was measured in the treated and untreated groups. (B and C) Representative kidney slices of untreated and treated groups groups after *K. pneumoniae* infection; total kidney histopathology scores. The arrows in the figure indicate histopathological characteristics, including tubular dilatation, tubular cell vacuolization/degeneration and spilling, glomerular atrophy or degeneration, dilatation in the Bowman’s capsule, and separation in the parietal layer of the

Bowman’s capsule. HE staining Scale bars = 50 μm (20×). Data are from three independent experiments. Statistically significant differences are indicated as follows: ns, no significance, * P<0.05, ** P<0.01, *** P<0.001, **** P<0.0001.

**Figure S4.** Construction of a mouse model of lung infection with *K. pneumoniae*. (A) The bacterial load in the lungs of mice was measured after 24 h infection with *K. pneumoniae* at different concentrations. (B) Kaplan-Meier survival plot of mice infected with *K. pneumoniae* at different concentrations. Statistically significant differences are indicated as follows: ns, no significance, * P<0.05, ** P<0.01, *** P<0.001, **** P<0.0001.


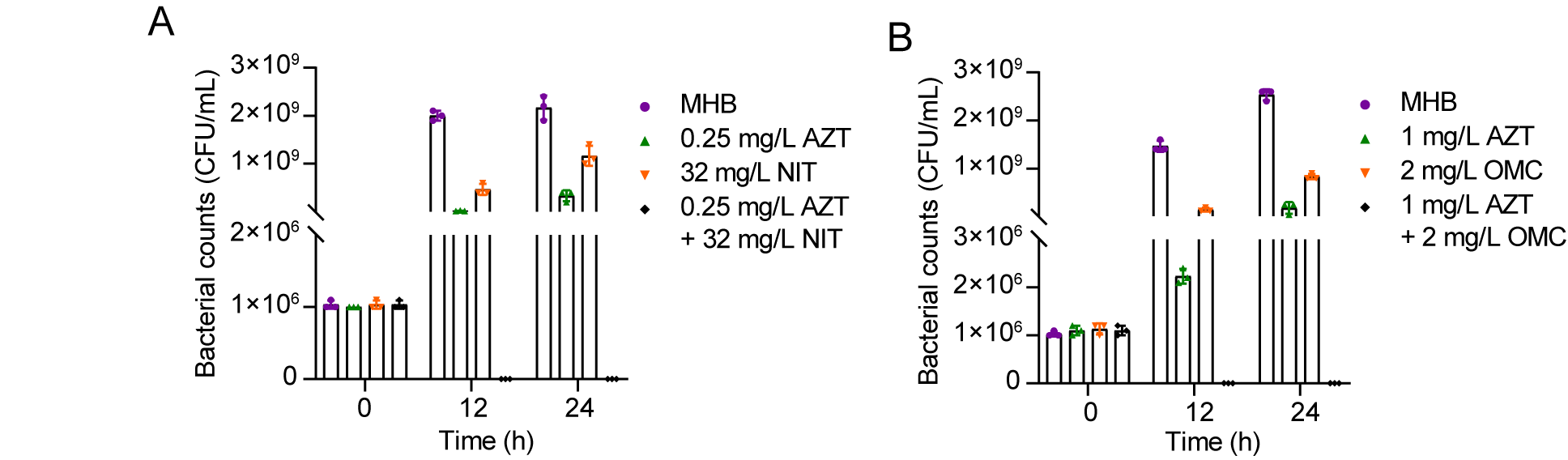


**Figure S1.** AZT combined with NIT or OMC significantly inhibited the growth of GN 172867. (**A**) Spot dilution assay. After 0, 12 and 24 h incubation at 37℃, serial 10-fold dilutions of GN 172867 containing MHB, 0.25 mg/L AZT or 32 mg/L NIT alone or in combination (AZT+NIT) were spotted onto MHA plates. (**B**) Spot dilution assay. After 0, 12 and 24 h incubation at 37℃, serial 10-fold dilutions of GN 172867 containing MHB, 1 mg/L AZT or 2 mg/L OMC alone or in combination (AZT+OMC) were spotted onto MHA plates. The plates were incubated overnight and then the colony counts were performed.. All experiments were performed three times, and the mean ± SD is shown.


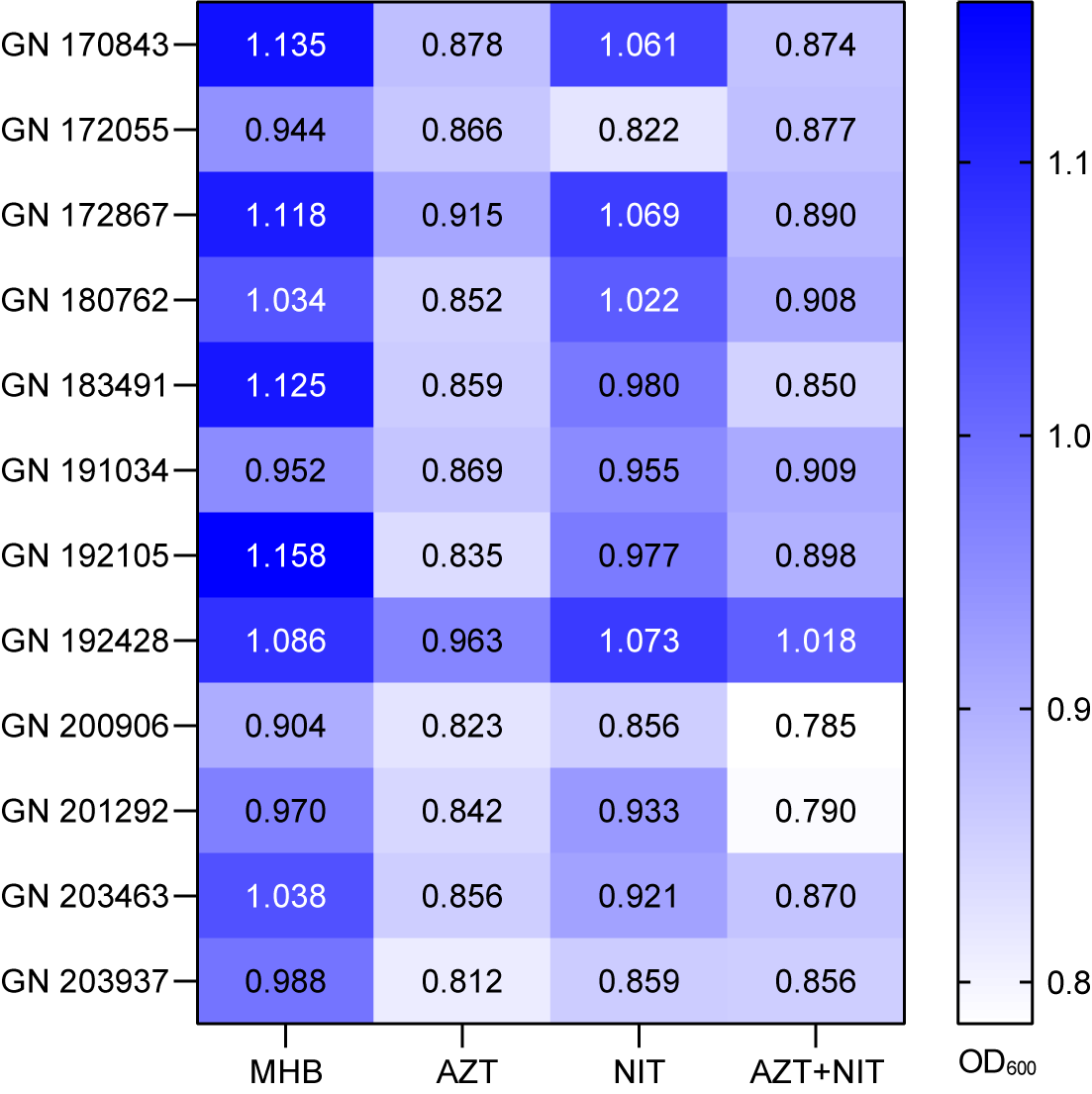


**Figure S2.** Twelve clinical MDR *K. pneumoniae* strains were treated with 0.125 mg/L AZT, 4 mg/L NIT, 0.125 mg/L AZT+4 mg/L NIT, or not for 36 h; planktonic cell growth was detected by OD_600_ analysis. Data are from three independent experiments.

**
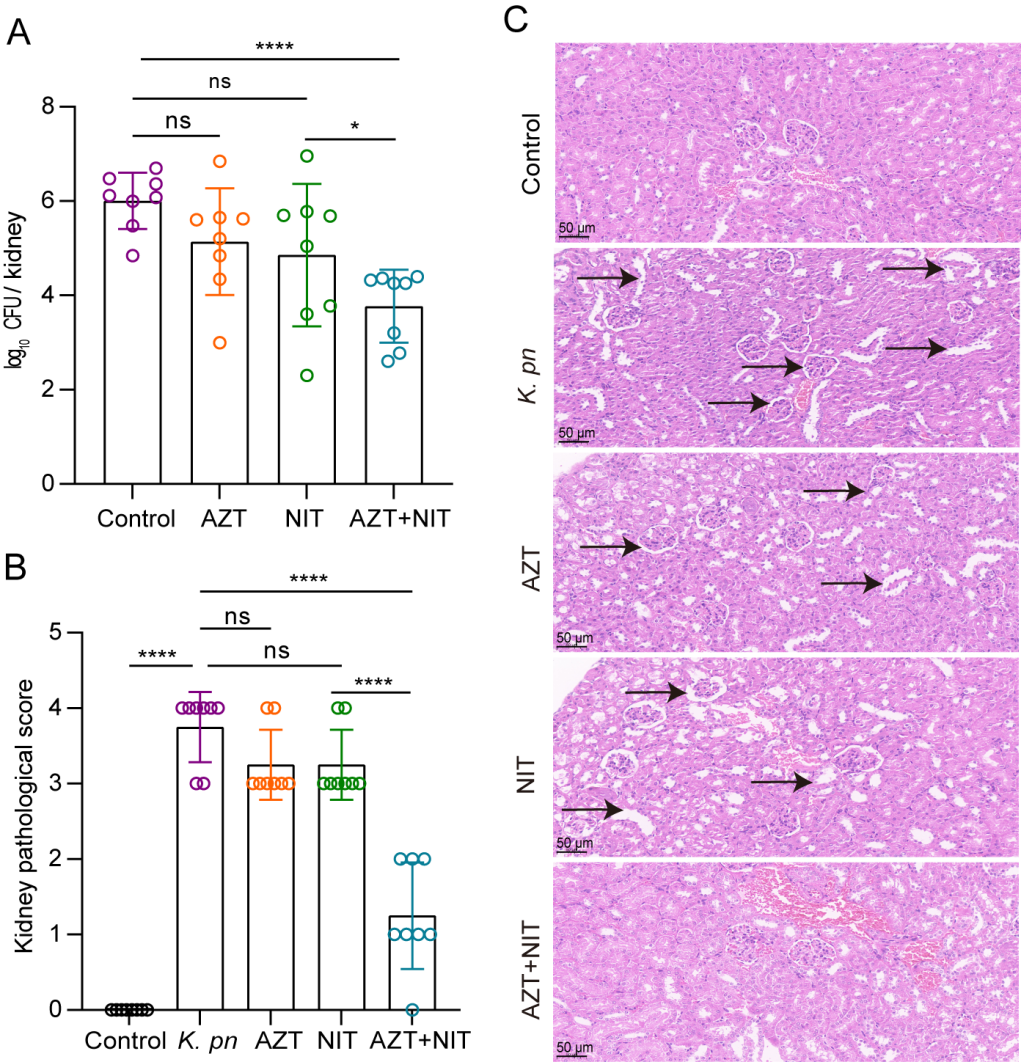
**

**Figure S3.** Effects of AZT and NIT on kidney in mice with urinary tract infection caused by *K. pneumoniae* GN 172867. (**A**) After 24 h of infection with *K. pneumoniae*, the kidney bacterial load was measured in the treated and untreated groups. (**B** and **C**) Representative kidney slices of untreated and treated groups groups after *K. pneumoniae* infection; total kidney histopathology scores. The arrows in the figure indicate histopathological characteristics, including tubular dilatation, tubular cell vacuolization/degeneration and spilling, glomerular atrophy or degeneration, dilatation in the Bowman’s capsule, and separation in the parietal layer of the Bowman’s capsule. HE staining Scale bars = 50 μm (20×). Data are from three independent experiments. Statistically significant differences are indicated as follows: ns, no significance, * P<0.05, ** P<0.01, *** P<0.001, **** P<0.0001.


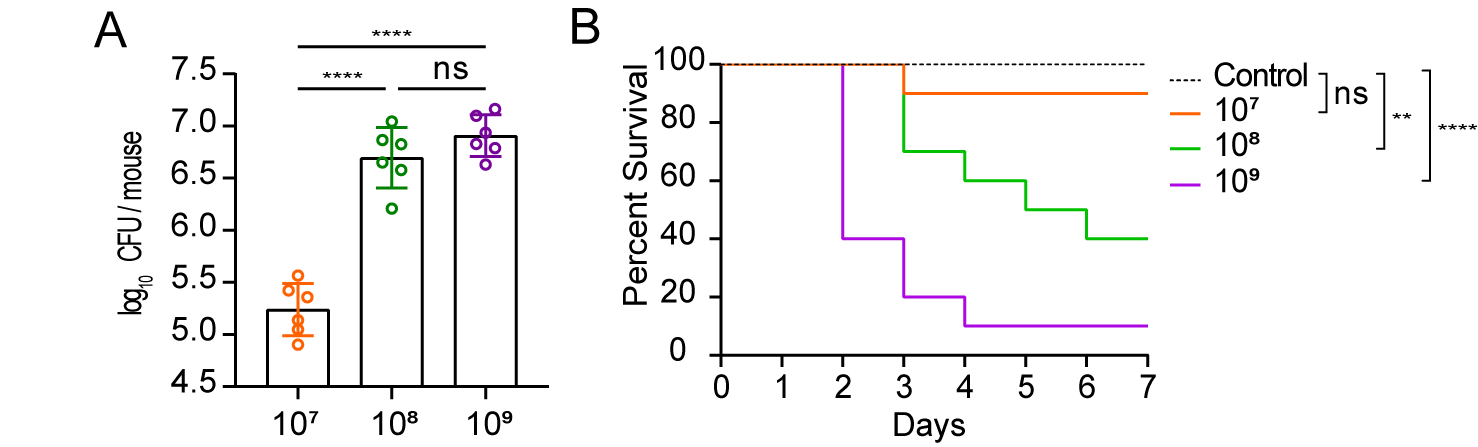


**Figure S4.** Construction of a mouse model of lung infection with *K. pneumoniae*. (**A**) The bacterial load in the lungs of mice was measured after 24 h infection with *K. pneumoniae* at different concentrations. (**B**) Kaplan-Meier survival plot of mice infected with *K. pneumoniae* at different concentrations. Statistically significant differences are indicated as follows: ns, no significance, * P<0.05, ** P<0.01, *** P<0.001, **** P<0.0001.
